# Omega-3–rich fish oil lipid emulsions prevent sepsis-driven mesenteric hypoperfusion and multiorgan injury in a murine model

**DOI:** 10.55730/1300-0144.6224

**Published:** 2026-05-16

**Authors:** Gökşen ÖZ, Esin ÖZÇELEBİ, Ayşe Yeşim GÖÇMEN, Cenk SÖKMENSÜER, Selma YILDIRIM, Arzu TOPELİ, Alper Bektas İSKİT

**Affiliations:** 1 Department of Medical Pharmacology, Ufuk University, Faculty of Medicine, Ankara, Turkiye; 2 Department of Medical Pharmacology, Hacettepe University, Faculty of Medicine, Ankara, Turkiye; 3 Department of Pathology, Hacettepe University, Faculty of Medicine, Ankara, Turkiye; 4 Division of Intensive Care Medicine, Department of Internal Medicine, Hacettepe University, Faculty of Medicine, Ankara, Turkiye

**Keywords:** Intensive care, sepsis, parenteral nutrition, lipid emulsions, omega-3 fatty acids, shock, fish oil

## Abstract

**Background/aim:**

Parenteral feeding is used in critically ill patients when enteral nutrition is inadequate or intolerable, or when the gastrointestinal tract is nonfunctional. Lipid emulsions play a major role in parenteral nutrition, supplying the necessary fatty acids and providing high-caloric density. We investigated the effects of various parenteral lipid emulsions on mesenteric perfusion, inflammation-induced oxidation, and organ damage in a lipopolysaccharide-induced mouse endotoxemia model.

**Materials and methods:**

Organ damage induced by lipopolysaccharide in septic mice was assessed histopathologically and biochemically, and an ultrasonic blood flowmeter was used to measure the impact of lipid emulsions on mesenteric blood flow

**Results:**

Lipopolysaccharide injection significantly reduced mesenteric blood flow when compared to saline controls (3.02 ± 0.10 mL/min vs 1.44 ± 0.10 mL/min, p < 0.05). Only fish oil emulsion treatment significantly abrogated this decrease (2.84 ± 0.39 mL/min, p < 0.05). None of the tested lipid emulsions prevented lipopolysaccharide-induced increases in liver and spleen weights. Fish oil reduced liver damage in septic mice, whereas specific dosages of olive and soybean oils caused significant histopathological liver and spleen damage even in the control animals. Furthermore, soybean and olive oil also significantly increased serum oxidative stress in the healthy control mice (2.55 ± 0.06 vs 4.93 ± 0.38 and 4.28 ± 0.12, p <0.05).

**Conclusion:**

Fish oil emulsions with high omega-3 fatty acid content abrogated the decrease in mesenteric blood flow and prevented liver damage in this experimental endotoxemia model.

## 1. Introduction

Parenteral feeding is used in critically ill patients with gastrointestinal dysfunction if enteral nutrition is not adequate. Lipid emulsions, which offer high caloric density and contain necessary fatty acids, constitute the foundation of parenteral nutrition and may also affect immunological response [1]. Soybean oil (SO), which contains n-6 polyunsaturated fatty acids (PUFA), is the primary component in conventional lipid emulsions. However, soybean-based lipid emulsions increase the availability of free arachidonic acid [2], the precursor fatty acid of inflammatory mediators, which are crucial for various molecular and cellular processes [3]. Newer lipid emulsions provide less n-6 fatty acids through the partial replacement of SO with oils that provide n-9 monounsaturated fatty acids or n-3 PUFA [4,5]. Studies analyzing the use of various parenteral lipid emulsions (PLEs) in sepsis and critical illness have produced contradictory results [6]; however, more recent PLEs have been found to have less impact on oxidative stress, immunological response, and inflammation [7,8].

Sepsis is the leading cause of morbidity and mortality in intensive care units (ICUs) [9] due to the potential for circulatory dysfunction and hypoperfusion, leading to multiorgan failure. There have been several studies to date investigating the clinical importance of oxidative stress in sepsis [10], and greater antioxidant activity has been reported in patients who recovered from sepsis than those who did not [11]. In two prospective observational studies, an association was observed between total antioxidant capacity and Acute Physiology and Chronic Health Assessment II (APACHE II) score, while antioxidant deficiency was linked to greater mortality [11, 12].

Nutritional guidelines provide limited and nonspecific recommendations regarding optimal lipid composition selection in patients with sepsis or those receiving parenteral nutrition [13,14]. The present study analyzes the effects of various PLEs on mesenteric perfusion, inflammatory oxidative stress, and organ histopathology in an experimental, LPS-induced, mouse endotoxemia model.

## 2. Materials and methods

### 2.1. Animals

48 Swiss albino mice (25–35 g) were obtained from the Experimental Animals Breeding Unit of the Refik Saydam Hygiene Center of the Turkish Republic Ministry of Health. All mice were acclimatized for two weeks before the experiment and kept under environmentally controlled conditions at 21 ± 2 °C and 30–70% relative humidity with a 12 h light/12 h dark cycle (lights were kept on between 07.00 and 19.00 h). Ad libitum access was provided to tap water (drinking bottle) and standard pellet l chow (Dokuz Tug Yem Sanayi, Ankara, Türkiye). All the procedures described within this manuscript were executed in strict accordance with the Guiding Principles in the Care and Use of Laboratory Animals . The study protocol was approved by the Institutional Experimental Animal Care and Use Ethics Committee of Hacettepe University (Approval Number: 2019/04-04) before the commencement of any intervention.

### 2.2. General procedure

Endotoxemia was induced in mice using endotoxin derived from *Escherichia coli* (lipopolysaccharide (LPS), O55:B5; 10 mg/kg in 10 mL/kg volume, i.p.)—a well-established model for systemic inflammation [15]. While LPS is commonly used to model sepsis-related inflammation, it can induce a reproducible state of endotoxemia, rather than polymicrobial sepsis [16, 17]. The mice were fasted overnight but were given ad libitum access to drinking water before the experiment. The animals were then anaesthetized with chloralhydrate (400 mg/kg, i.p.), and a 1 cm midline incision was made. The control group received saline (1 mL) subcutaneously rather than PLE immediately after surgery to produce the hyperdynamic phase of sepsis. After the LPS injection, the animals were placed in separate cages to recover from the interventions under standard conditions in the Laboratory Animal Husbandry Facility of Hacettepe University, Faculty of Medicine, Department of Pharmacology.

### 2.3. Experimental protocol

After 4 h of LPS or saline injection, the mice were given fish oil (FO) (Omegaven), SO (Kabiven), olive oil (OO) (Oliclinomel) (50 mL/kg, s.c.; in 1 mL; n = 6 for each group) or nonpyrogenic sterile saline (NaCl 0.9%, w/v, dissolved in pyrogen-free distilled water; s.c., in 1 mL, n = 6). Lipid emulsions were administered subcutaneously to model continuous parenteral delivery during the hyperdynamic phase of sepsis. The same route was used for the control animals that received saline to ensure experimental consistency.

Omegaven is a FO-based lipid emulsion containing highly refined FO (10.0 g), eicosapentaenoic acid (1.25–2.82 g), docosahexaenoic acid (1.44–3.09 g), and α-tocopherol (0.015–0.0296 g) per 100 mL. Kabiven, derived from SO, is a combination of amino acids, electrolytes, dextrose, and soya lipids (20.0 g per 100 mL). Oliclinomel N4-550E is a lipid emulsion mixture of 80% refined OO (16.0 g per 100 mL) and 20% refined soya oil (4.0 g per 100 mL), some amino acids, and glucose.

**Figure 1:**
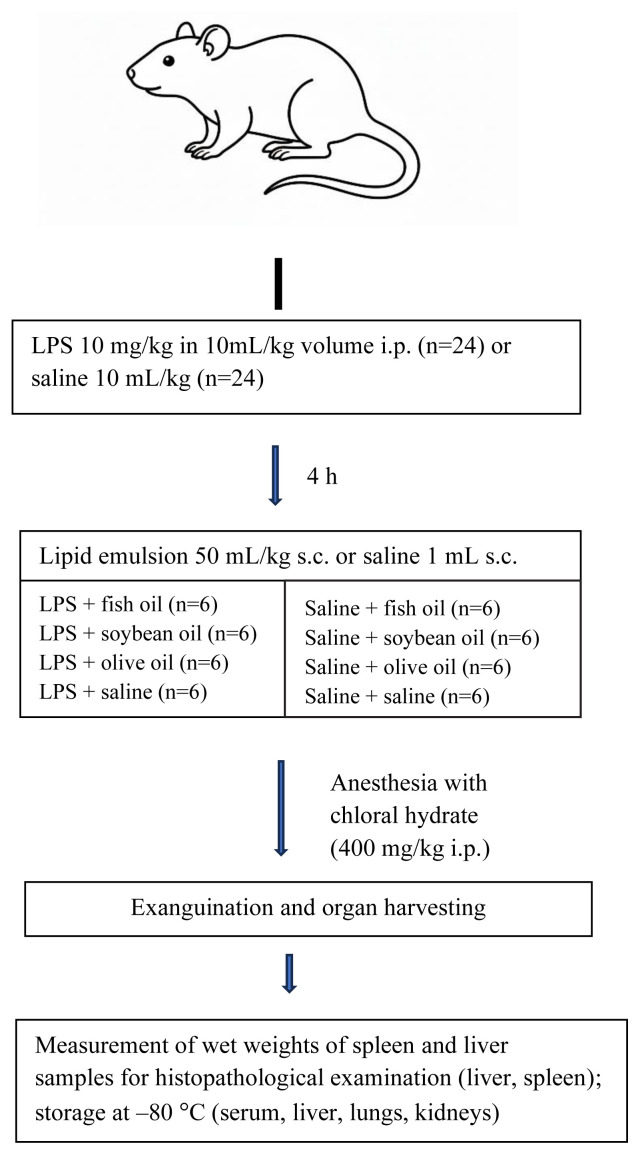
Experimental protocol.

All emulsions were prepared daily and warmed to body temperature (approximately 37 °C) before being injected. The emulsions were kept in dark containers until the injections to protect them from light-induced decomposition. The experimental groups and the detailed chronological sequence of the procedures are summarized in Figure 1.

### 2.4. Surgical procedure

The basic surgical procedures used in the present study were previously described [18]. The mice were anaesthetized with chloral hydrate (400 mg/kg, i.p.) at the 4th h following LPS injection and then placed on a heat-insulated cork-sheet-covered operating table. The animals were allowed to breathe room air spontaneously. The body temperatures of the mice were kept at 37.0 ± 0.1 °C using a rectal thermistor probe-controlled incandescent lamp (100W) placed approximately 30 cm above the animals.

A midline incision was made, and a perivascular ultrasonic Doppler-flow probe, connected to a Transonic Small Animal Flowmeter System T106 (Transonic, Ithaca, New York, USA) was placed around the common mesenteric artery. Absolute blood flow values were measured in milliliters per minute for 15 min following the procedure detailed in the author’s previous publications [16]. The values were then normalized for each mouse by dividing by the individual animal’s body weight and were expressed as mL/min/kg body weight. Flowmeter values were also recorded on a computer using an MP35 Biopac data recording system (Goleta, California, USA).

After mesenteric blood flow was measured for 15 min, the animals were euthanized by severing the common carotid artery. Both kidneys were then quickly removed, together with the spleen, lung, and liver, and placed on hydrophilic paper tissue to remove any remaining fluid. The wet weights of the spleen and the liver were measured. Approximately half of each liver sample was separated and stored at –80 °C for biochemical analysis, while the rest of the organs were placed in formaldehyde fixation for conventional light microscopic examination. The entire procedure (from exsanguination to tissue fixation) was completed within 2 min.

### 2.5. Histological examination

The spleen and half of the liver samples were fixed in 10% buffered formaldehyde and processed using routine light microscopic tissue processing techniques, and 5 μm sections were stained with hematoxylin-eosin and examined and photographed with an Olympus BH-2 (Japan) conventional light microscope. The liver and spleen tissues of each animal were subjected to histological analysis. Two to three slides were prepared for each organ, with six animals per group examined in a blinded manner.

### 2.6. Biochemical analyses

Total antioxidant status (TAS) and total oxidant status (TOS) measurements were made from tissue homogenates and serum using commercial ELISA kits (Rel Assay Kit Diagnostics; Gaziantep, Türkiye) according to the manufacturer’s instructions. The oxidative stress index (OSI = TOS/ TAS) was calculated, and the data were presented as OSI.

### 2.7. Drugs and reagents

Sodium chloride and hematoxylin were purchased from Merck (USA). Lipopolysaccharide (E. coli endotoxin, serotype O55:B5) and eosin were obtained from Sigma (USA). Paraffin was purchased from Shandon (UK), and formaldehyde was obtained from Carlo Elba (Italy).

An SO-based emulsion (Kabiven, Fresenius), an OO-based emulsion (Oliclinomel, Baxter), and an FO-based emulsion (Omegaven, Fresenius Kabi) were used in the study. These commercially available PLEs are widely used clinically in Türkiye and worldwide.

### 2.8. Statistical analyses

Normally distributed continuous data are presented as mean ± standard error of the mean (SEM). The SEM was chosen to highlight the estimated precision of the group means. Statistical analyses were made with one-way analysis of variance (ANOVA) or with a Kruskal–Wallis test for independent groups, as appropriate. Dunn’s Multiple Comparison Test was used for post hoc analyses. The sample size was determined based using the resource equation method to ensure statistically significant results. Statistical analyses, calculations, and graphical presentations were created using the Microsoft Office Excel and GraphPad 6.0 programs. p-values of <0.05 were considered statistically significant.

## 3. Results

### 3.1. Effects of lipid emulsions on mesenteric blood flow in mice

**Figure 2:**
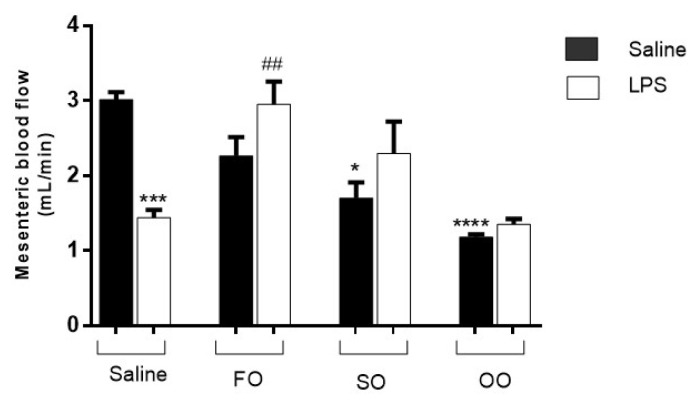
Effects of PLE on mesenteric blood flow (mL/min) values in the saline and LPS-treated groups (*used to show statistical significance compared to the saline-saline group, # used to show statistical significance compared to the PS-saline group; * p < 0.05, ** p < 0.01, *** p < 0.001, **** p < 0.0001). FO: fish oil, SO: soybean oil, OO: olive oil.)

Mesenteric blood flow was statistically significantly decreased in the LPS (endotoxin) group compared to the control group (mL/min; saline: 3.02 ± 0.10, LPS: 1.44 ± 0.10, n = 12, p <0.05; Figure 2). Mesenteric blood flow was significantly higher in the LPS-FO group than in the LPS group (mL/min; LPS-FO: 2.84 ± 0.39, p <0.05).

### 3.2. Effect of lipid emulsions on liver and spleen weights

**Figure 3:**
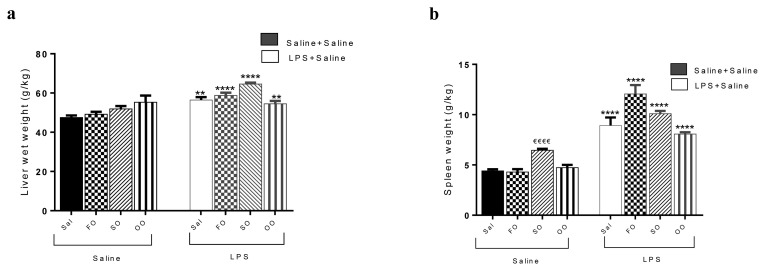
Effects of PLEs on liver (a) and spleen (b) wet weight/body weight ratio (per kg body weight; g/kg) in saline- and LPS-treated mice. * indicates statistical significance compared to the saline–saline group (**** p < 0.0001). Effects of PLEs on liver wet weight/body weight ratio (per kg body weight; g/kg) in saline- and LPS-treated mice. € indicates significance within the saline group according to Kruskal–Wallis analysis; saline–saline and saline–SO subgroups show statistical significance within the group (€€€€ p < 0.0001). Sal: saline, FO: fish oil, SO: soybean oil, OO: olive oil.

After the mesenteric blood flow measurement and exsanguination procedures, the wet weights of the liver and spleen (g) per body weight (kg) were measured. Endotoxin increased liver weights (g per kg body weight: saline: 47.51 ± 1.05, endotoxin: 56.46 ± 1.46, n = 6, p <0.05; Figure 3a) and spleen (g per kg body weight: saline: 4.41 ± 0.17, endotoxin: 8.92 ± 0.80, n = 6, p <0.0001; Figure 3b). Lipid emulsions did not affect the increase in liver and spleen wet weight after LPS injection, while soybean oil increased the spleen weight compared to the control group (g per kg body weight: saline: 4.41 ± 0.17, SO: 6.45 ± 0.15, n = 6, p <0.0001; Figure 3b).

### 3.3. Liver, spleen, lung, kidney, and serum oxidative stress index

**Figure 4:**
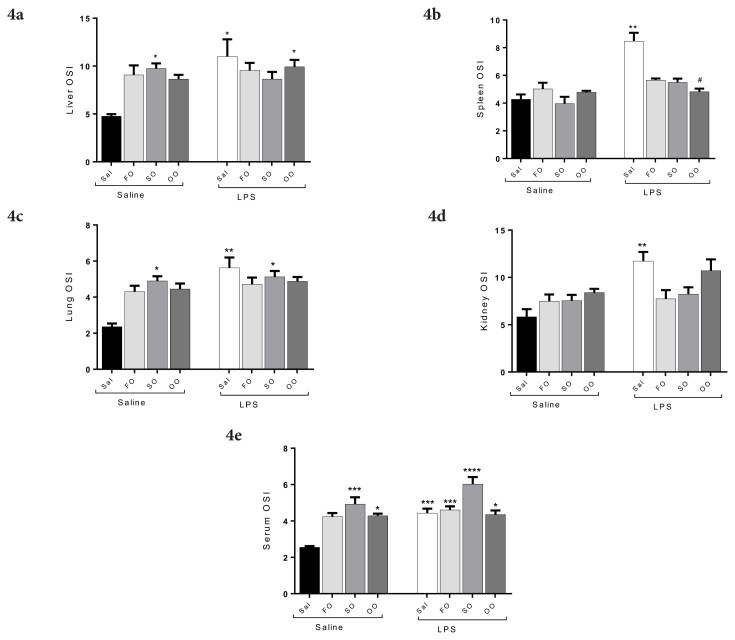
Effects of PLE on oxidative stress index (OSI) in mouse liver (a), spleen (b), lung (c), kidney tissues (d), and serum (e). (*used to show statistical significance compared to the saline-saline group, # used to show statistical significance compared to the LPS-saline group; * p < 0.05, ** p < 0.01, *** p < 0.001, **** p< 0.0001). FO: fish oil, SO: soybean oil, OO: olive oil.)

OSI was increased in the liver, kidney, lung, spleen, and serum after LPS challenge. SO increased hepatic OSI compared with the control group (saline-saline) (saline: 4.76 ± 0.23, SO: 9.75 ± 0.54, n = 6, p <0.05; Figure 4a). Olive oil significantly reduced the OSI in the spleen in the LPS group (LPS: 8.47 ± 0.61, OO: 4.82 ± 0.23, n = 6, p < 0.05; Figure 4b). SO in the lung increased the OSI, and the difference was statistically significant when compared to the control group (saline-saline) (saline: 2.36 ± 0.18, SO: 4.89 ± 0.26, n = 6, p < 0.05; Figure 4c). LPS-induced OSI levels in the kidney remained elevated, with no significant difference observed following lipid emulsion treatment (saline: 5.84 ± 0.80, LPS: 11.72 ± 0.98, n = 6, p < 0.05; Figure 4d). SO and OO significantly increased the OSI in the serum compared to the control group (saline-saline) (saline: 2.55 ± 0.06, SO: 4.93 ± 0.38, OO: 4.28 ± 0.12, n = 6, p < 0.05; Figure 4e).

### 3.4. Histopathological effects of lipid emulsions on organ damage

**Figure 5:**
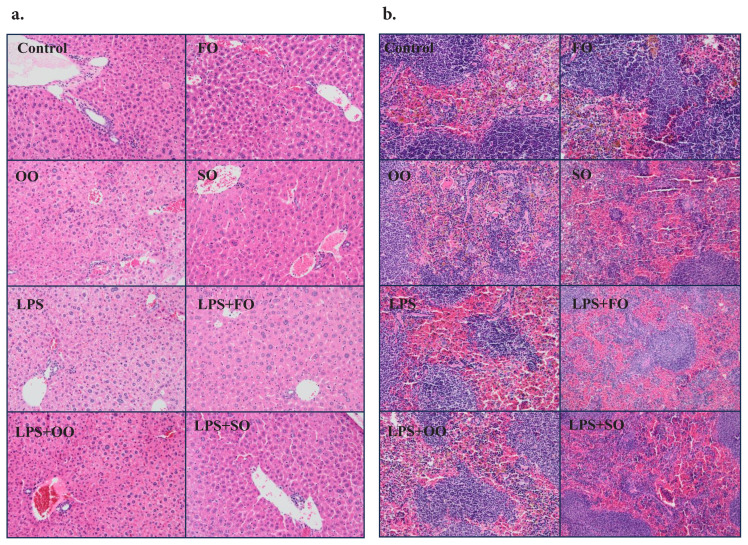
a) Liver histopathology of saline and LPS-treated groups with different PLE. b) Spleen histopathology of saline and LPS-treated groups with different PLEs.

LPS challenge induced significant injury in both liver and spleen tissues on histopathological evaluation (Figures 5a and 5b). In the control group, the administration of lipid emulsions alone was associated with varying degrees of baseline histological changes, including portal inflammation and hepatocellular injury (Table). While FO treatment partially mitigated LPS-induced hepatic damage when compared with the LPS-alone group, no such effects were observed with the soybean and OO treatments. Congestion was the primary histological finding in the spleen across all groups, and no restorative effects were observed with any of the lipid emulsions tested.

## 4. Discussion

Our findings demonstrate that among the PLEs tested, FO provides a distinct protective advantage in the presence of endotoxemia. Specifically, FO was the only intervention capable of reversing LPS-induced mesenteric hypoperfusion and mitigating hepatic histological damage. While SO and OO are widely utilized in clinical practice, their effects in this acute endotoxemia model were less favorable, often failing to counteract the oxidative and structural damage triggered by LPS.

Sepsis induces hypoperfusion in specific organ and tissue beds to maintain perfusion of vital organs [19,20]. Splanchnic hypoperfusion is clinically significant, and relative hypoperfusion dramatically exacerbates systemic inflammation in patients with gastrointestinal injury alongside severe sepsis. The clinical consequences of the early interruption of cutaneous and mesenteric circulation in sepsis have raised academic interest in mesenteric blood flow [21,22]. The relative hypoperfusion and disruption of mesenteric circulation in the gastrointestinal system allows bacteria to enter systemic circulation (bacterial translocation) and restimulate inflammation pathways by disrupting intestinal mucosal integrity [23].

In the present study, only FO significantly improved blood flow in the mesenteric artery associated with sepsis. All lipid emulsions, including FO, reduced blood flow in the control group, and the decline was statistically significant with OO. FO was essential for increasing blood flow in sick animals. Mesenteric hypoperfusion is thought to be associated with vasoconstriction resulting from the release of endogenous catecholamines and endothelin. In one study, vasoconstriction was attenuated by the endothelin receptor antagonist bosentan [24]. Monitoring mesenteric blood flow and vascular resistance is thus critical for predicting the outcome of septic shock. Lipid emulsions containing FO may be effective in the treatment of recognized septic shock and may affect prognosis by inhibiting many of the diseases that occur after mesenteric hypoperfusion and that worsen prognoses. In our experimental study, FO was noted to improve mesenteric hypoperfusion, and so should be investigated further.

An increase in the wet liver and spleen weight is an expected finding after LPS application in experimental septic shock models [24]. However, none of the PLEs in the present study prevented this increase in weight.

Histopathological analysis corroborated the hemodynamic findings. LPS-induced parenchymal destruction and inflammatory infiltration in the liver were significantly attenuated only in the group treated with FO, while SO and OO had no such similar restorative effects on liver architecture. The antioxidant properties of OO have been described in several in vitro and long-term in vivo studies [25,26]; however, in our acute model, OO was noted to significantly increase serum OSI and failed to provide protection against renal oxidative stress. This suggests that the high doses or the rapid administration required in acute experimental settings may override the potential benefits of OO’s phenolic components.

The assessment of TAS and TOS in this experimental septic shock model revealed that LPS mediated significant oxidative damage. LPS reduces protective TAS levels and increases TOS levels and has been identified as causing an increase in reactive oxygen species, lipid peroxidation products, superoxide anion, peroxides, and malondialdehyde (MDA). Each of these components is critical to the biology and outcome of sepsis [27,28]. Septic shock occurs when active neutrophils produce reactive oxygen species (ROS) and enzymes such as myeloperoxidase (MPO) [29]. Antioxidants maintain the balance between ROS production and elimination, and ROS clearance is impaired in sepsis due to associated endothelial damage and multiorgan failure. In contrast to the histological examination, TAS and TOS measurements were performed on serum, kidney, and lung samples rather than liver and spleen tissues due to the possible release of chemicals from the lungs and kidneys that may prevent oxidative damage. It was noted, however, that none of the parenteral lipid emulsions reduced oxidative damage in the liver. OO significantly reduced OSI in the spleen in the LPS group, which is consistent with previous studies [30], and SO in the mouse lung increased OSI in the saline group, which was also consistent with earlier studies [31,32]. The LPS-induced oxidative stress in the kidney could not be mitigated by the applied lipid emulsions. The serum results are the most striking element of these assays, as OO increased the OSI in normal animal serum considerably, like SO. At this dose, all PLEs decreased protective serum TAS concentrations in the control group. While there are numerous studies and in vitro research reporting the antioxidant properties of OO, such an in vivo outcome is intriguing [7,25,30]. These studies show that, with few exceptions, PLEs do not protect against oxidative damage. It has been demonstrated in normal, healthy animals that such doses cause oxidative damage to the lungs and decrease antioxidant substances in serum. It is worth noting that even in saline-treated animals, certain emulsions, such as SO, increased OSI values, suggesting that these lipids may exert oxidative effects independently of inflammatory stimuli.

While earlier studies have emphasized the cardiovascular benefits of dietary fats, more recent research suggests that OO-based PLEs may be advantageous in critical care due to their antioxidant properties, although knowledge of the influence on sepsis and septic shock is limited. The cardioprotective effects of the Mediterranean diet are largely attributed to OO phenolics, including hydroxytyrosol and oleuropein [31, 32]. The many biological activities of OO phenolics include suppression of LDL oxidation associated with the onset of atherosclerosis, inhibition of platelet aggregation, reduced LTB4 and TXB2 secretion from activated leukocytes, scavenging of superoxide and other reactive oxygen radicals, increased plasma antioxidant capacity, and enhanced nitric oxide release from macrophages [33]. In general, these beneficial effects are associated with long-term uses of OO, however, its use for the treatment of lung injury in animal models via intravenous or direct injection into the lungs warrants further study [34]. In the present study, parenteral OO was administered intraperitoneally—a route similar to that used in animal models of lung injury.

Inflammatory response and oxidant capacity were examined in a study of MCT-LCT, SO-OO, and SO-OO-FO formulations as lipid sources for postoperative parenteral feeding in patients following major abdominal surgery [35]. The authors reported that a blend of 20% SO and 80% OO greatly reduced OXLDL3 (Oxidized LDL3) levels while increasing TBARS (Thiobarbituric Acid Reactive Substance) levels significantly. OXLDL3 and TBARS are less accurate than the TAS and TOS measurements employed in this study, and TBARS, in particular, is a somewhat outdated assay used to assess plasma antioxidant capability and lipid peroxidation levels. In our investigation, OO induced organ damage in control mice and did not protect against LPS effects. The large dose of LPS we utilized, as well as the high dose of OO emulsion, may have had a negative impact on this outcome.

SO-containing emulsions are long-chain triglycerides (LCT) used in many enteral and parenteral formulations. Corn oil and sunflower oil also present similar properties to SO and contain LCT. With these oils, essential fatty acid deficiency can be prevented. However, they contain high amounts of polyunsaturated fatty acids and 52–54% linoleic acid [36]. After the regular infusion of soybean-derived emulsions, a decrease in plasma lipoprotein levels of α-tocopherol and a decrease in antioxidant capacity were observed. Being the oldest lipid emulsion, this group is used by mixing OO and SO at a ratio of 80/20 or enriched with α-tocopherol. In the present study, PLE containing SO and OO did not prevent LPS damage, and the protective effect of OO was observed only in the spleen, while oxidative damage in the lung and serum was increased.

There are several limitations of this study that warrant consideration. First, the 4-h observation window focuses strictly on the acute phase of injury, and so the long-term metabolic consequences or actual survival benefits of these emulsions remain unknown. Second, the doses of PLEs used, while based on experimental protocols, may exceed typical clinical infusion rates, which may explain the baseline histological and oxidative changes observed in healthy controls. The lack of a survival analysis in the study can be considered another limitation.

## 5. Conclusion

FO-based lipid emulsions, characterized by high omega-3 fatty acid content, show significant potential in preserving mesenteric perfusion and hepatic structural integrity during the early, acute stages of septic shock. FO may ameliorate early-stage endotoxemia-associated damage, potentially via the modulation of inflammatory and oxidative responses. All of the lipid emulsions, including FO, can cause organ damage in healthy animals, even in protective doses. The results of the present study suggest that the correct choice of lipid components in parenteral nutrition is vital for the modulation of the inflammatory and oxidative trajectory of critically ill patients.

## Figures and Tables

**Table:** Histopathological scores of liver and spleen. The effects of fish oil, soybean oil, and olive oil applications on the liver and spleen of control and LPS groups (n = 6/group; 2–3 slides per organ per animal were evaluated). LPS: lipopolysaccharide, Sal: saline, FO: fish oil, SO: soybean oil, OO: olive oil.

Group		LIVER						SPLEEN		

		Portal Inflammatory Reaction	Intrasinusoidal Congestion	Intrasinusoidal Lymphoreticular Reaction	Hepatocellular Injury	Ballooning Degeneration	SCORE	Congestion	Capsulitis	SCORE

**Control**	Sal	0	0	0	0	0	**0**	0		**0**
	FO	1	1	1	1	2	**6**	1		**1**
	SO	1	1	0	1	0	**3**	2		**2**
	OO	1	1	0	2	2	**6**	1		**1**
									
**LPS**	Sal	1	1	0	1	2	**5**	1		**1**
	FO	2	0	1	0	0	**3**	2	**+**	**2**
	SO	1	1	1	2	1	**6**	2	**+**	**2**
	OO	1	2	0	1	2	**6**	1		1
